# PGK1 Drives Glial Glycolytic Reprogramming to Mediate Isoflurane‐Induced Cognitive Impairment in Aged Mice

**DOI:** 10.1111/jcmm.71276

**Published:** 2026-07-02

**Authors:** Zhuo Ren, Huaying Hu, Jiahao Guo, Yajun Liu, Chenyang Qiu, Kai Yuan, Yujie Ma, Shuntao Jiao, Ruiyun Guo, Dengfa Zhao, Dongliang Zhang, Yang Zeng, Jun Ma, Mianwang He

**Affiliations:** ^1^ Department of Obstetrics and Gynecology Beijing Chao‐Yang Hospital, Capital Medical University Beijing China; ^2^ Medical Innovation Research Division Chinese PLA General Hospital Beijing China; ^3^ Beijing Jiaen Hospital Beijing China; ^4^ School of Life Sciences and Technology Shandong Second Medical University Weifang Shandong China; ^5^ Department of Orthodontics Beijing Stomatological Hospital, Capital Medical University School of Stomatology, Capital Medical University Beijing China; ^6^ Institute of Hematology, Fifth Medical Center of Chinese PLA General Hospital Beijing China; ^7^ Hebei Technology Innovation Center for Stem Cell and Regenerative Medicine Shijiazhuang Hebei China; ^8^ Human Anatomy Department Hebei Medical University Shijiazhuang Hebei China; ^9^ Department of Neurology Chinese PLA General Hospital Beijing China

**Keywords:** cognitive impairment, glycolysis, microglia, neuroinflammation, PGK1

## Abstract

Isoflurane‐induced neuroinflammation triggers cognitive impairment in aged mice, but its underlying mechanism remains unclear. This study investigated the molecular mechanism by which isoflurane promotes glycolytic reprogramming to cause cognitive dysfunction in aged mice and identified potential therapeutic targets. 18‐month‐old mice were placed in an anaesthetic induction chamber containing 2% isoflurane (ISO) for 2 h to induce anaesthesia; this procedure was repeated daily for 5 days to establish a model of age‐related cognitive impairment in mice. Behavioural studies in mice were conducted using the Y‐Maze, contextual fear conditioning test (CFCT), Novel Object Recognition (NOR) test, and water maze test. Immunofluorescence analysis was employed to detect changes in the expression of microglia‐related proteins PGK1, Hip, iNOS, Arg1, and P65. RT‐PCR was employed to detect changes in mRNA expression levels of GLUT2, PKM2, HK2, and LDHA, which are associated with cellular metabolic reprogramming. ELISA was used to measure alterations in TNF‐β, IL‐4, IL‐10, IL‐6, IL‐1β, and TNF‐α cytokines. ISO enhanced glycolytic flux via PGK1, thereby driving microglia polarization toward the pro‐inflammatory M1 phenotype and triggering neuroinflammation, ultimately leading to cognitive impairment in mice. Supplementing the glycolytic intermediate FBP reversed the anti‐inflammatory effects induced by PGK1 knockdown, confirmed that PGK1 exerted its effects through the “PGK1‐glycolysis axis.” Mechanistically, PGK1 knockdown effectively suppressed M1 polarization of microglia while promoting their transition to the anti‐inflammatory M2 phenotype. This significantly mitigated ISO‐induced neuroinflammation and neuronal injury, ultimately improving cognitive function in mice. These findings reveal that PGK1 serves as a key molecular link between ISO anaesthesia and neuroinflammatory cognitive impairment. Targeting and inhibiting PGK1 exerts neuroprotective effects by reprogramming microglial glucose metabolism and phenotype, providing novel theoretical insights and potential therapeutic strategies for preventing ISO‐induced neurological complications.

## 1 Introduction

Isoflurane (ISO)‐induced cognitive impairment, a common neurological complication, remains poorly understood in terms of its underlying mechanisms [1]. Research indicates that cognitive impairment may be associated with mechanisms such as neuroinflammatory responses, neuronal apoptosis, and energy metabolism abnormalities [2, 3, 4]. Concurrently, fundamental and clinical neuroscience studies suggest that postoperative cognitive impairment is more prevalent among elderly patients, with a portion progressing to dementia within 3–5 years [5]. With the global aging population, the number of elderly patients undergoing surgical procedures continues to rise. ISO‐induced nerve damage not only severely impacts patients' long‐term quality of life and postoperative recovery but also imposes a significant socioeconomic burden. Preclinical studies indicate that the inhalation anaesthetic ISO can trigger central nervous system inflammation, inducing cognitive deficits in elderly animal models that mimic the clinical manifestations of human cognitive impairment [6, 7].

Microglia, as the brain's innate immune cells, not only perform immune surveillance functions but also play a crucial role in maintaining central nervous system homeostasis and promoting repair [8, 9, 10]. Furthermore, microglial dysfunction may contribute to the chronic neuroinflammatory processes underlying neurodegenerative diseases [11, 12]. Research indicates that ISO activates microglia, inducing their polarization toward a pro‐inflammatory M1 phenotype, which leads to the release of large amounts of pro‐inflammatory cytokines and subsequent neuronal damage [13]. However, the upstream metabolic regulatory mechanisms driving the M1 polarization of microglia under ISO exposure remain incompletely understood. Similar metabolic conversion mechanisms have been reported in Alzheimer's disease and LPS‐activated microglia [14, 15]. Diseases associated with neuroinflammation, such as Alzheimer's disease and Parkinson's disease, are closely linked to alterations in cellular glycolysis. Whole‐brain metabolic assessments indicate that a significant decline in glucose consumption rates may correlate with the onset of early‐stage Alzheimer's disease [16]. Furthermore, studies have demonstrated that LPS stimulation of microglia enhances anaerobic glycolysis and inhibits mitochondrial oxidative phosphorylation, further confirming metabolic dysfunction in microglia under pathological conditions [15, 17]. Excessive glycolysis within cells leads to increased lactate production, causing accumulation of reactive oxygen species that attack neuronal lipids, proteins, and DNA, thereby resulting in neuronal damage. However, the role of microglia in glycolysis during ISO‐induced cognitive dysfunction remains unclear.

PGK1 is a key protein kinase in glycolysis with a molecular weight of 44.6 kDa, catalysing the conversion of ADP to ATP [18]. Studies indicate that PGK1 expression is elevated in diseases such as cancer, neurological disorders, and diabetes, exacerbating disease damage [19, 20, 21]. Li et al. discovered that PGK1 suppresses mitochondrial pyruvate utilization by promoting PDHK1 phosphorylation, thereby reducing reactive oxygen species (ROS) production in tumour cells [18]. Multiple studies have confirmed that PGK1 promotes tumorigenesis by accelerating glycolysis [22, 23, 24]. Recent research indicates that PGK1 not only promotes M1 macrophage polarization via NLRP3 but also regulates the role of glycolysis in enhancing M1 polarization and inflammation in microglia [25]. Additionally, studies have shown that PGK1 is a key point in the metabolic defects of neurons caused by Parkinson's disease [26]. However, the specific role of PGK1 in ISO‐induced cognitive impairment in aged mice remains to be elucidated.

This study employed ISO to establish a mouse model of cognitive impairment, investigating the role of PGK1 in glucose metabolism dysfunction and neuroinflammation in mice. The findings suggest that PGK1 may serve as a potential key therapeutic target for treating ISO‐induced cognitive impairment and other neuroinflammation‐related disorders.

## 2 Methods and Materials

### 2.1 Materials

ISO (PHR2874) was purchased from Sigma‐Aldrich; LPS (L2630) was purchased from Sigma‐Aldrich; The glucose assay kit (S0201S), L‐lactic acid assay kit (S0208S), and ATP assay kit (S0026) were all purchased from Beyotime; glucose (D9434) was purchased from Sigma‐Aldrich. The CCK‐8 assay kit was purchased from MedChemExpress. In Situ Cell Death Detection Kit, TMR red (12156792910) was purchased from Roche. Nissl staining solution (C0117) was purchased from Beyotime. SteadyPure RNA Extraction Kit (AG21024) and Evo Super M‐MLV One‐Step RT‐PCR Mix (AG11624) were purchased from Accurate Biology.IL‐10, IL‐4, IL‐6, IL‐1β ELISA assay kits were purchased from Thermo Fisher Scientific. TNF‐α and TNF‐β ELISA assay kits were purchased from BioLegend. Anti‐PGK1 (ab199438), anti‐NeuN (ab190195), anti‐Iba1 (ab283319), and anti‐Arg1 (ab96183) were purchased from Abcam. Anti‐HIP (#2723), Anti‐iNOS (#13120), and Anti‐p65 (#4764) were purchased from Cell Signalling Technology.

### 2.2 Animals and Drug Administration

Male C57BL/6J mice aged 18 months were purchased from Changzhou Cavens Laboratory Animal Company. During the rearing period, mice had free access to water and food. The temperature was maintained at 22°C–23°C, with a 12 h light/dark cycle. During the ISO modelling procedure, mice were placed in an anaesthesia induction chamber containing 2% ISO and subjected to daily anaesthesia for 2 h over 5 consecutive days and conduct behavioural testing on the fifth day following the completion of the ISO treatment [27, 28]. All experimental procedures were approved by the Animal Welfare and Ethics Committee of Beijing MDKN Biotechnology Co. Ltd. and were strictly conducted in accordance with the Beijing Animal Control Committee's Guidelines for the Care and Use of Laboratory Animals (MDKN‐2024‐210).

### 2.3 Morris Water Maze (MWM) Tests

Use food‐grade titanium dioxide to whiten the water, adjusting the temperature to 22°C–25°C. Submerge the platform in the water, ensuring it remains 1 cm below the surface. First, conduct an adaptive swimming test for 1–3 min. Then place the mouse on the platform for 20 s to complete the preliminary training. During the formal experiment, place the mouse facing the pool wall and release it into the water from one of the four quadrants. Monitor the mouse to prevent drowning. A video recording system was used to track the swimming trajectories of mice. Each mouse swam for 60 s. Mice that failed to climb onto the platform within 60 s were manually guided onto the platform and remained there for 10 s. This training was conducted for 4 consecutive days. Following training, the platform was removed. Mice were gently placed into the water from the quadrant opposite the platform and allowed to swim freely for 60 s. Each mouse's swimming trajectory and the number of times it crossed the platform location were observed and recorded. Data were automatically logged using the ANY‐maze behavioural tracking software.

### 2.4 Y‐Maze

The Y‐Maze consists of three arms of equal length with adjacent angles of 120°, designated as the novel arm, start arm, and other arm. A movable barrier is placed at the entrance of the novel arm. The duration mice spend in the novel arm is analysed to assess their spatial memory function. Prior to the experiment, all three arms are disinfected with 75% alcohol and allowed to air‐dry before testing commences. First, place the mouse in the start arm for 10 min of free exploration as an adaptation training session. 1 h after the training concludes, formally commence the experiment. Open the barrier of the novel arm, place the mouse at the bottom of the start arm, and set the observation time to 5 min. Finally, the ANY‐maze detection software will automatically identify, record, and perform the final result analysis.

### 2.5 Novel Object Recognition (NOR)

The experiment consists of two phases. First, place two blue cubes in the fixed environment, ensuring both objects maintain an equal distance from the chamber walls. Place the mouse in the fixed environment for 10 min of adaptation training. Remove the mouse and allow a 1 h interval. Then, replace one of the cubes in the fixed environment with a green sphere (different colour, shape, and volume) and position it in the same location. Reintroduce the mouse to the fixed environment for 5 min. Record the activity process of mice recognizing and exploring novel objects in a fixed environment. Using a video capture and analysis system, document the time and frequency of mice's nose tips or forelimbs entering the green sphere or blue cube regions. Recognition Index = Time spent exploring new objects / (Time spent exploring new objects + Time spent exploring familiar objects) × 100%. After completing the experiment with each mouse, disinfect the area with 75% alcohol, allow it to air dry, and then place the next mouse. Maintain a quiet environment and constant temperature throughout the experiment.

### 2.6 Contextual Fear Conditioning Test (CFCT)

On the first day, mice underwent habituation training by being placed in the fear cage with the entrance and exit closed for 10 min. On the second day, mice were reintroduced to the same fear cage used for habituation. After 2 min of free activity, they received auditory and foot shock stimuli. Each shock lasted 2 s at 0.6 mA, with 1 min intervals between shocks. On the third day, mice were placed in the fear cage and exposed to the same auditory stimulus used during training for 10 min. The freezing time ratio of the experimental mice was recorded and analysed using software.

### 2.7 Nissl Staining

Perfuse mice with 4% PFA, remove tissues, fix overnight, dehydrate with graded alcohol solutions, clear with xylene, then embed in paraffin. Section at 8 μm thickness and heat‐mount slides. Place sections in xylene for dewaxing, hydrate with graded ethanol, then rinse with ddH_2_O. stained with toluidine blue solution in a 60°C constant‐temperature drying oven for 10 min, rinsed with ddH_2_O, and decolorized in 95% ethanol. After decolorization, samples were thoroughly dehydrated in 100% ethanol, cleared in xylene, mounted with neutral resin, and examined under an optical microscope.

### 2.8 Immunofluorescence Staining

Perform whole‐body perfusion of mice with 4% PFA. Remove tissues and fix overnight. Embed in OCT and section frozen tissue into 5 μm‐thick slices. Permeabilize tissue with 0.05% Triton X‐100 for 15 min, block with 10% goat serum for 1 h. Incubate overnight at 4°C with primary antibodies (Hip, NeuN, Iba1, PGK1, Arg1, iNOS, p65) according to the manufacturer's instructions. Wash three times with PBS. Incubate with secondary antibodies at room temperature for 1 h. Wash three times with PBS. Stain with DAPI for 5 min. Wash three times with PBS. Mount with an antifade mounting medium. Acquire images using a confocal microscope. Analyse data using ImageJ software.

### 2.9 Real‐Time Polymerase Chain Reaction (RT‐PCR)

Extract RNA from hippocampal tissue using an RNA extraction kit. Add the RNA template, primers, and reaction mix to PCR tubes according to the RT‐PCR kit instructions, then perform the PCR reaction under the conditions specified in the manual. The relevant primer sequences (Table 1) are as follows:

**TABLE 1:** Primer sequences of RT‐PCR.

Gene	Forward (5′ → 3′)	Reverse (5′ → 3′)
iNOS	TGAACTACGTCCTGTCCCCT	CTCTTCTCTTGGGTCTCCGC
IL‐1β	TGGCAACTGTTCCTG	GGAAGCAGCCCTTCATCTTT
Arg1	CAGAAGAATG GAAGAGTCAG	CAGATATGCAGGGAGTV
CD206	CAGGTGTGGGCTCAGGTAGT	TGTGGTGAGCTGAAAGGTGA
GLUT2	TCAGAAGACAAGATCACCGGA	GCTGGTGTGACTGTAAGTGGG
PKM2	CTATCCTCTGGAGGCTGTGC	CCAGACTTGGTGAGGACGAT
HK2	GAGCCACCACTCACCCTACT	CCAGGCATTCGGCAATGTG
LDHA	CAAAGACTACTGTGTAACTGCGA	TGGACTGTACTTGACAATGTTGG
β‐Actin	GTCCCTCACCCTCCCAAAAG	GCTGCCTCAACACCTCAACCC

### 2.10 Primary Microglia Culture and Transfection

We prepared primary microglia from newborn P3 wild‐type C57 mice. Cells were cultured in DMEM/F12 medium supplemented with 10% FBS, 1% penicillin–streptomycin solution, nitrogen sources, and macrophage colony‐stimulating factor (M‐CSF). After 14 days of culture, microglia were harvested for subsequent experiments. After overnight culture of primary microglia, shRNA‐PGK1 lentiviruses were introduced (Genepharma, China). Phagocytosis assays were performed 48 to 72 h post‐transfection.

### 2.11 Enzyme‐Linked Immunosorbent Assay (ELISA)

Following the kit instructions, extract the hippocampal tissue supernatant and dilute it. Proceed according to the reagent supplier's protocol, briefly outlined below. Add the test samples and standards to the pre‐coated microplate wells. Incubate at 37°C for 1 h. Wash away the liquid from the wells. Add the corresponding biotin‐labelled detection antibody. Incubate again at 37°C for 1 h. Wash away the excess liquid from the wells to remove unbound detection antibody. Add streptavidin‐HRP to each well and incubate at room temperature in the dark for 1 h. Add TMB or other colorimetric substrate solution and incubate in the dark for 30 min until a distinct gradient of blue appears in the high‐concentration standard wells. Add stop solution and immediately measure the absorbance at 450 nm using a microplate reader.

### 2.12 Western Blotting

Proteins were extracted from hippocampal tissue using RAPI. Protein quantification was performed with the BCA assay kit. Proteins were separated by SDS‐PAGE and transferred to PVDF membranes. Membranes were blocked with TBST at room temperature for 1 h. Primary antibodies specific to the target protein were added and incubated overnight at 4°C. After washing with TBST, appropriate secondary antibodies were added. Following another TBST wash, membranes were developed using ECL chemiluminescent reagent.

### 2.13 Cell Culture and Reagents

HT22 cells were obtained from Procell Life Science & Technology Co. Ltd. Cells were cultured in Dulbecco's modified Eagle's medium (DMEM; Gibco, Grand Island, NY, USA) supplemented with 10% fetal bovine serum (FBS; Gibco), 100 U/mL penicillin, and 100 U/mL streptomycin. Cultures were maintained at 37°C in a humidified incubator with an atmosphere of 5% CO_2_. Prior to experimentation, cells were transduced with lentiviral particles expressing sh‐PGK1 (Hanbio Biotechnology Co. Ltd., Shanghai, China) carrying the target sequence. At 24 h post‐transduction, the medium was replaced with fresh complete medium. To establish an in vitro inflammatory injury model, cells were stimulated with 1 μg/mL LPS for 24 h [29].

### 2.14 CCK‐8 Assay

Cells in the logarithmic growth phase were seeded at a density of 2000 cells per well into a 96‐well plate and cultured for 24 h. A 10 μL volume of CCK‐8 reagent was added to each well, incubated in the incubator for 2 h, and the absorbance was measured at 450 nm.

### 2.15 TUNEL Assay

Cultivate well‐growing cells in confocal culture dishes for 24 h. Fix with 4% paraformaldehyde for 15 min, then treat samples with PBS containing 1% Triton X‐100 for 15 min. Directly add a mixture of TdT enzyme and fluorescein‐labelled dUTP to the samples. Incubate at 37°C in the dark for 1 h. Wash away excess dye with PBS. Counterstain nuclei with DAPI. Mount slides with an anti‐fluorescence fading mounting medium. Acquire images using a confocal microscope.

### 2.16 Glycolytic Stress Test

After seeding well‐viable cells into microplate wells and completing the initial setup, inject glucose into each well at 3 h, oligomycin at 6 h, and 2‐DG at 10 h. Monitor ECAR continuously throughout the entire experiment.

### 2.17 Glucose Detection Experiment

Prepare a glucose standard solution of known concentration according to the kit instructions. First, dispense 5 μL of sample or standard into a 96‐well plate. Add 185 μL of Glucose Assay Reagent to achieve a final volume of 190 μL, then centrifuge to settle the liquid at the bottom of the well. Heat at 95°C for 8 min, then cool to 4°C. Measure the absorbance at 630 nm using an enzyme‐linked immunosorbent assay reader.

### 2.18 Lactic Acid Detection Test

Prepare a known concentration of lactic acid standard solution and WST‐8 Working Solution according to the kit instructions. Dispense 50 μL of each sample into the sample wells of a 96‐well plate, including a blank control well. Add 50 μL of WST‐8 Working Solution to each well. Mix thoroughly and incubate at 37°C in the dark for 30 min. Measure the absorbance at 450 nm using a microplate reader.

### 2.19 ATP Detection Test

Add 200 μL of ATP lysis buffer to each well of a 6‐well plate. After lysis, centrifuge at 45,000 rpm for 5 min and collect the supernatant. Prepare the ATP standard curve and working detection solution according to the manual. Add 100 μL of ATP working detection solution to each well of a 96‐well plate and let stand at room temperature for 5 min. Then add 20 μL of sample or standard to each well. After 2 s, immediately measure the RLU value using a luminometer.

### 2.20 Statistical Analyses

Data are expressed as mean ± standard error of the mean (SEM). Comparisons among multiple groups were performed using one‐way analysis of variance (ANOVA), while comparisons between two groups were conducted using the *t*‐test. Statistical analyses were performed using GraphPad Prism 9.5.1 software (San Diego, California, USA), with a significance level set at *p* < 0.05.

## 3 Results

### 3.1 ISO Exposure Induces Multidimensional Cognitive Deficits in Mice

**FIGURE 1:**
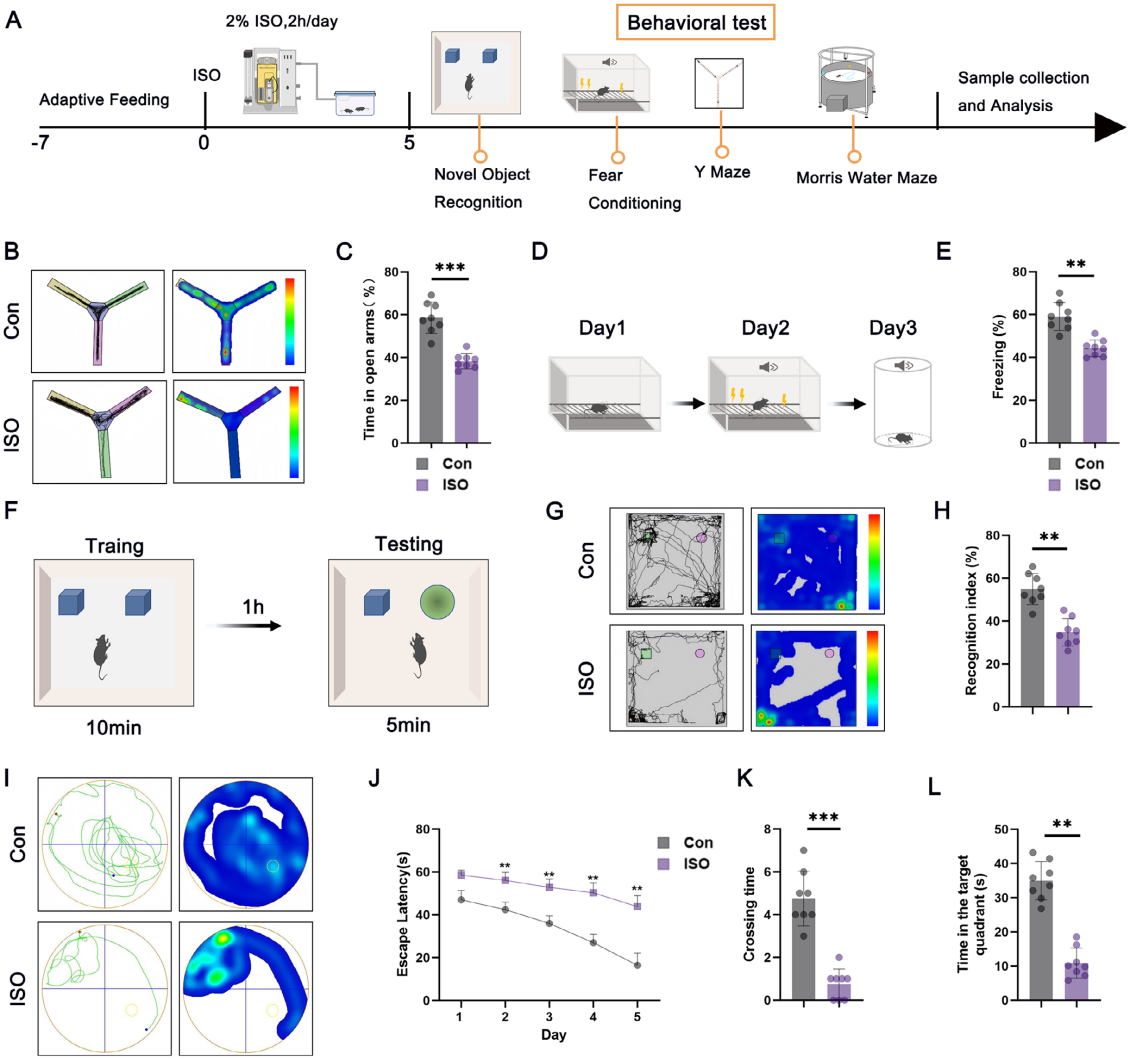
ISO Exposure Induces Multidimensional Cognitive Deficits in Mice. (A) Experimental Design Flowchart. (B, C) Mouse Y‐Maze and Statistics. (D, E) Mouse contextual fear conditioning test (CFCT). (F) Mouse novel object recognition (NOR) experiment. (G) NOR movement trajectories. (H) NOR index statistics. (I) MWM mouse swimming trajectories. (J) MWM mouse escape latency. (K) MWM mouse platform crossing frequency statistics. (L) MWM mouse dwell time in target quadrant. *n* = 8, **p < 0.01, ***p < 0.001.

Prior to formal experiments, mice underwent 1 week of acclimation. Subsequently, mice were anaesthetised daily with 2% ISO for 2 h over five consecutive days. Behavioural testing commenced on day 6, followed by relevant analyses upon completion (Figure 1A). We assessed ISO's effects on mouse cognitive function using the Y‐Maze, CFCT, NOR, and MWM. The Y‐Maze evaluated spatial memory, revealing significantly reduced time spent in the novel arm in the ISO group compared to the Control group (Figure 1B,C), indicating impaired spatial memory. Additionally, the CFCT experiment assessed contextual memory. Results showed a significantly reduced freezing time ratio in the ISO group compared to the Control group (Figure 1D,E), indicating impaired contextual memory. NOR was used to assess the mice's ability to distinguish objects. Results showed that compared to the Control group, mice in the ISO group spent significantly more time exploring novel objects and significantly less time exploring familiar objects (Figure 1F,G). Further statistical analysis indicated that the ISO group exhibited a significantly lower discrimination index than the Control group (Figure 1H), suggesting that ISO significantly impaired the mice's object recognition memory. MWM results showed that ISO group mice exhibited significantly longer immobility times compared to the Control group (Figure 1I). Preliminary MWM training results showed that compared to the Control group, mice in the ISO group exhibited significantly prolonged latency to reach the platform from day 2 to day 5, indicating differences in learning and memory abilities (Figure 1J). To assess spatial memory retention, the platform was removed post‐training, and the number of platform crossings and dwell time in the target quadrant were quantified across groups. Results revealed significantly reduced platform crossings and target quadrant dwell time in the ISO group compared to the Control group, indicating impaired memory retention (Figure 1K,L). These findings suggest that ISO induces cognitive dysfunction in mice.

### 3.2 PGK1 Upregulation Mediates ISO‐Induced Microglial Inflammation and Synaptic Damage

**FIGURE 2:**
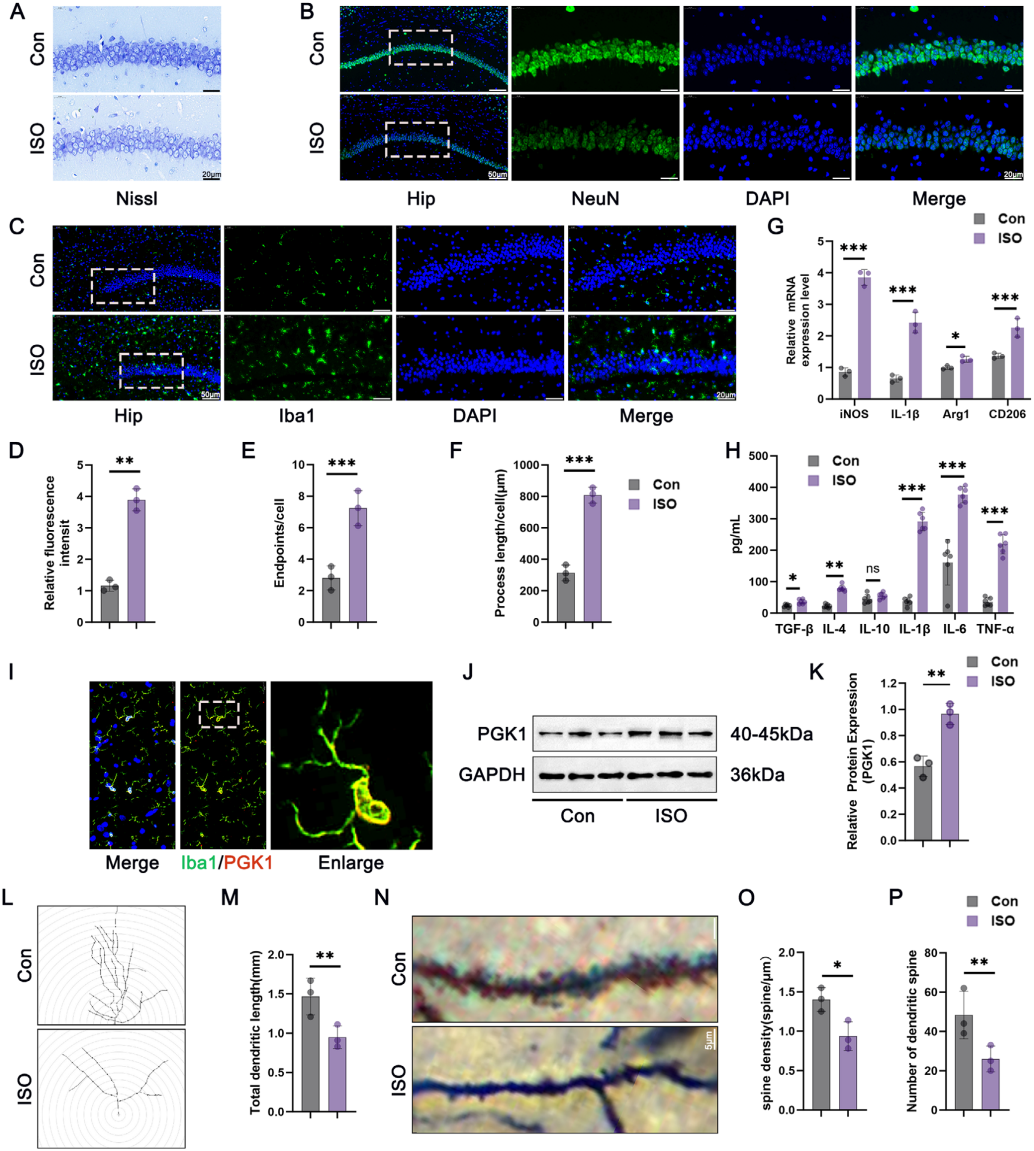
PKG1 upregulation mediates ISO‐induced microglial inflammation and synaptic damage. (A) Nissl‐stained microglia, scale bar = 20 μm. (B) Representative images of co‐localized immunofluorescence staining for microglia Hip and NeuN, scale bar = 20 μm. (C) Representative images of co‐localized immunofluorescence staining for microglia Hip and IBA1, scale bar = 20 μm. (D) Relative fluorescence intensity of Iba1. (E) Number of cell processes in individual microglia in panel C. (F) Total length of all branches in individual microglia in panel C. (G) RT‐PCR detection of mRNA expression levels for iNOS, IL‐1β, Arg1, and CD206 in the hippocampal region. (H) ELISA detection of cytokine expression for TNF‐β, IL‐4, IL‐10, etc., in the hippocampal region. (I) Representative immunofluorescence image of PKG1 in microglia. (J, K) Western blot analysis of PKG1 expression in microglia. (L) Sholl analysis of hippocampal neurons. (M) Quantitative analysis of dendritic complexity from Sholl analysis. (N) Representative images of dendritic spines in hippocampal neurons, scale bar = 5 μm. (O) Quantitative analysis of dendritic spine density. (P) Quantitative analysis of total dendritic spine number. *n* = 3, *p < 0.05, **p < 0.01, ***p < 0.001.

Neuronal damage underlies cognitive impairment in mice. Nissl staining revealed morphological changes in neurons: Control group neurons exhibited dense packing, plump cell bodies, and abundant dark Nissl bodies within the cytoplasm. In contrast, ISO‐induced damage resulted in loosely arranged neurons, partially shrunken cell bodies, and a significant reduction in Nissl bodies within the cytoplasm, which also exhibited lighter staining (Figure 2A). Immunofluorescence staining with NeuN to label hippocampal neurons revealed strong NeuN signals and weak Iba1 signals in the Control group. In contrast, the ISO group exhibited significantly reduced NeuN signals and markedly enhanced Iba1 signals compared to the Control group (Figure 2B–D). This indicates neuronal damage and heightened immune response following ISO stimulation. Next, we performed statistical analysis of microglial morphology. Results showed that compared with the control group, microglia in the ISO group exhibited significantly enlarged cell bodies, shorter processes, and significantly reduced total branch length. These morphological changes suggest their potential involvement in the release of cellular inflammatory cytokines (Figure 2E,F). RT‐PCR results revealed significantly elevated mRNA expression levels of iNOS, IL‐1β, Arg1, and CD206 in ISO‐treated mice compared to the Control group, indicating markedly increased intracellular inflammatory levels following ISO stimulation (Figure 2G). Furthermore, EILSA results demonstrated that, compared to the Control group, intracellular levels of inflammatory cytokines including TNF‐β, IL‐4, IL‐10, IL‐6, IL‐1β, and TNF‐α were significantly elevated following ISO treatment (Figure 2H). Immunofluorescence revealed colocalization of IBA1 and PGK1, confirming that PGK1 is expressed by microglia in tissues (Figure 2I). Subsequently, we detected PGK1 expression in microglia via Western blot and found significantly elevated PGK1 expression in microglia following ISO injury (Figure 2J,K). Given the unique structural complexity of neurons, we employed Sholl analysis to examine dendritic architecture across experimental groups. The results demonstrated that normal neurons exhibited intricate dendritic branching with a total dendritic length of approximately 1.5 mm. In contrast, ISO‐treated neurons showed significantly reduced dendritic complexity and a total length shortened by approximately one‐third compared to the Control group, suggesting that ISO induces structural impairment of dendrites that compromises neuronal function (Figure 2L,M). Finally, immunostaining revealed that ISO treatment significantly reduced both the density and number of dendritic spines compared to the Control group (Figure 2N–P), suggesting an impairment in neuronal information‐processing capacity. Collectively, these findings indicate that ISO induces neuronal structural damage and synaptic loss by activating microglia, triggering neuroinflammation, and upregulating PGK1 expression.

### 3.3 PGK1 Exacerbates Neuroinflammation by Driving Glycolytic Reprogramming and Impairing Energy Metabolism

**FIGURE 3:**
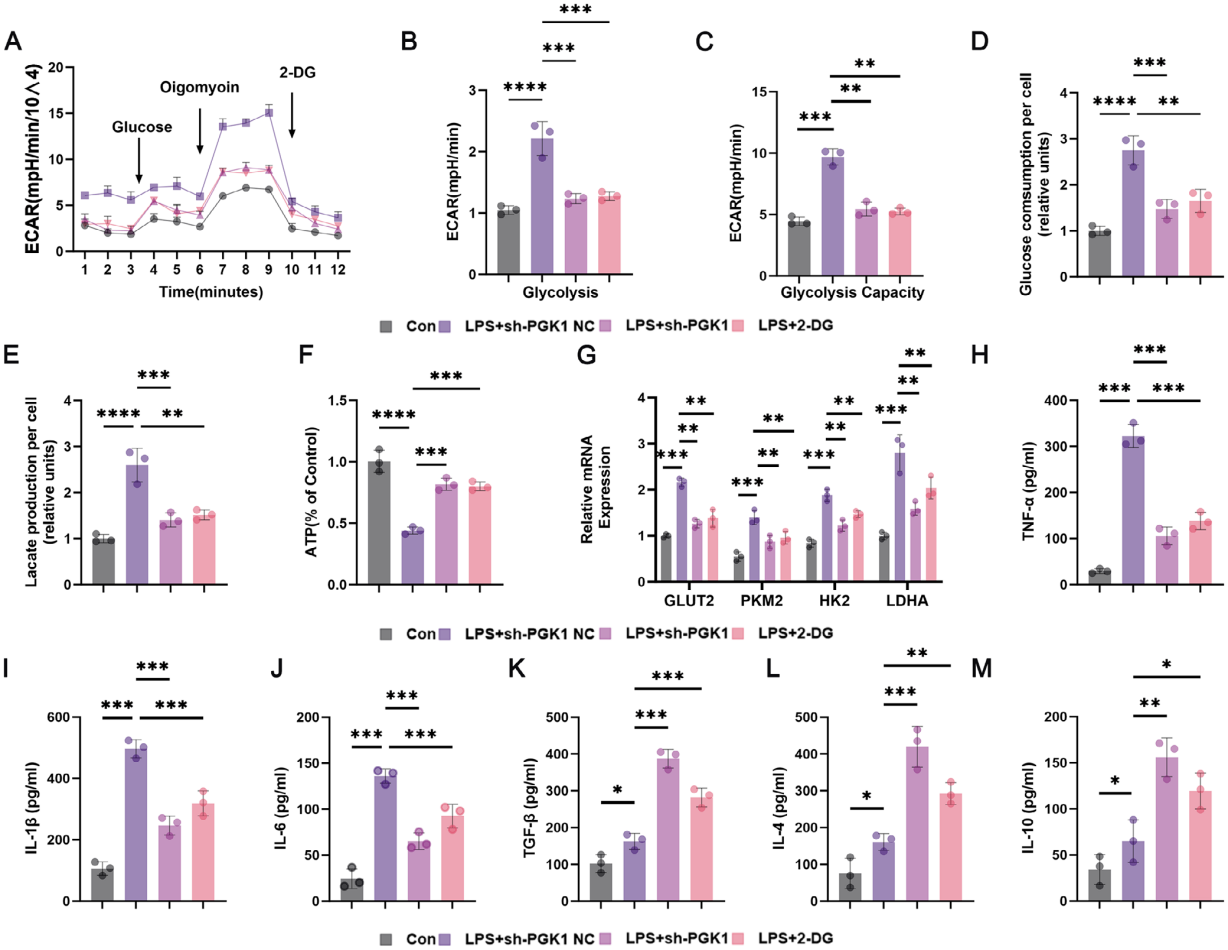
PGK1 exacerbates neuroinflammation by driving glycolytic reprogramming and impairing energy metabolism. (A) Results of the glycolytic stress assay. (B) Results of the Seahorse XF Cell Mito Stress Test. (C) ATP levels in cells from each group. (D) RT‐PCR analysis of M1 pro‐inflammatory iNOS and IL‐1β mRNA expression levels in cells from each group. (E) RT‐PCR analysis of M2‐type anti‐inflammatory Arg1 and CD206 mRNA expression levels in cells from each group. (F) RT‐PCR analysis of glycolysis‐related enzyme mRNA expression levels (GLUT2, PKM2, HK2 and LDHA) in cells from each group. (G, H) ELISA analysis of cytokine expression (TNF‐α, IL‐β, IL‐6, etc.) in cells from each group. (I) RT‐PCR analysis of mRNA expression levels of M1 and M2 pro‐inflammatory genes in cells from each group. (J) ELISA analysis of cytokine expression (including IL‐β, IL‐6, IL‐4, TGF‐β, IL‐10 and TNF‐α) in cells from each group. *n* = 3, *p < 0.05, **p < 0.01, ***p < 0.001.

To comprehensively evaluate the role of PGK1 in cellular glycolysis, we employed a glycolytic stress test supplemented with metabolite detection. The glycolytic stress test revealed that the basal glycolytic capacity and glycolytic reserve in the LPS + shPGK1 NC group were significantly higher than those in the Control group. This indicates that cells in this group rely more heavily on glycolysis for energy under steady‐state conditions and can mobilize a stronger glycolytic response under stress. Conversely, reducing PGK1 expression markedly decreased basal glycolytic function and glycolytic capacity in the LPS + shPGK1 NC group, suggesting that PGK1 downregulation may partially reverse abnormal glycolytic metabolism (Figure 3A). ORC curve analysis and ATP content measurements revealed that in the LPS group, mitochondrial basal respiration, ATP‐coupled respiration, and maximum respiratory capacity were all significantly reduced; in the LPS + sh‐PGK1 group, impaired mitochondrial respiratory function was significantly restored, as evidenced by an increase in basal OCR, enhanced ATP‐synthesis‐related respiration, and a significant improvement in maximum respiratory capacity. In contrast, in the LPS + 2‐DG group, there was no significant improvement in indicators related to mitochondrial respiration, suggesting that PGK1‐mediated enhancement of glycolysis is a key factor in LPS‐induced mitochondrial respiratory dysfunction in microglia (Figure 3B,C). RT‐PCR results indicated that downregulation of PGK1 suppressed LPS‐induced mRNA levels of the pro‐inflammatory cytokines iNOS and IL‐1β, whilst promoting an increase in the mRNA levels of the anti‐inflammatory factors Arg1 and CD206 (Figure 3D,E). Furthermore, downregulation of PGK1 significantly reduced the mRNA levels of glycolysis‐related enzymes GLUT2, PKM2, HK2, and LDHA following LPS stimulation, suggesting that PGK1 may be a key protein involved in cellular metabolic reprogramming (Figure 3F). ELISA results indicate that reducing PGK1 suppresses LPS‐induced increases in cellular TNF‐α, TNF‐β, IL‐1β, IL‐6, IL‐4, and IL‐10 levels, suggesting that PGK1 may inhibit cellular inflammatory responses (Figure 3H–M). RT‐PCR results showed that downregulation of PGK1 reduced the mRNA levels of the pro‐inflammatory factors iNOS and IL‐1β following LPS stimulation, whilst promoting the expression of the anti‐inflammatory factors Arg1 and CD206. Exogenous administration of TNF‐α partially reversed this effect, whereas the NF‐κB inhibitor SC75741 exhibited an inhibitory effect similar to that of PGK1 downregulation (Figure 3I); ELISA results further demonstrated that LPS‐stimulated cells simultaneously secrete pro‐inflammatory factors (IL‐1β, IL‐6) and anti‐inflammatory factors (IL‐4, TGF‐β). Knockdown of PGK1 significantly inhibited the release of pro‐inflammatory factors and promoted the release of anti‐inflammatory factors, whilst also promoting IL‐10 secretion and inhibiting TNF‐α secretion; exogenous TNF‐α partially reversed these changes, and SC75741 also exhibited a similar inhibitory effect (Figure 3J). These findings suggest that PGK1 may influence the balance of cytokine secretion in microglia by regulating LPS‐ and TNF‐α‐related pathways.

Next, we further validated the role of PGK1 in mediating ISO‐induced microglial dysfunction. Morphological analysis revealed that ISO stimulation significantly increased the number of cell endpoints and total process length per cell in primary microglia (Figure S1A,B). The results of the glycolysis level detection experiment showed that PGK1 knockdown or 2‐DG treatment effectively attenuated ISO‐induced elevations in glucose consumption, lactate production, and glycolytic capacity (Figure S1C–F), suggesting PGK1 as a key regulator of microglial metabolic reprogramming. Finally, triple‐immunofluorescence was employed to determine the cellular specificity of PGK1 expression in vivo. While ISO + LPS treatment significantly upregulated PGK1 MFI in Iba1+ microglia (Figure S1K,L), no significant changes were observed in NeuN+ neurons or GFAP+ astrocytes (Figure S1G–J). These findings demonstrate that the ISO‐induced upregulation of PGK1 is specifically restricted to the microglial population. Collectively, the above results indicate that PGK1 exacerbates neuroinflammation by driving the reprogramming of glycolysis and impairing energy metabolism.

### 3.4 Targeting PGK1 Suppresses Inflammation and Promotes Neuronal Survival by Inducing M2 Polarization in Microglia

**FIGURE 4:**
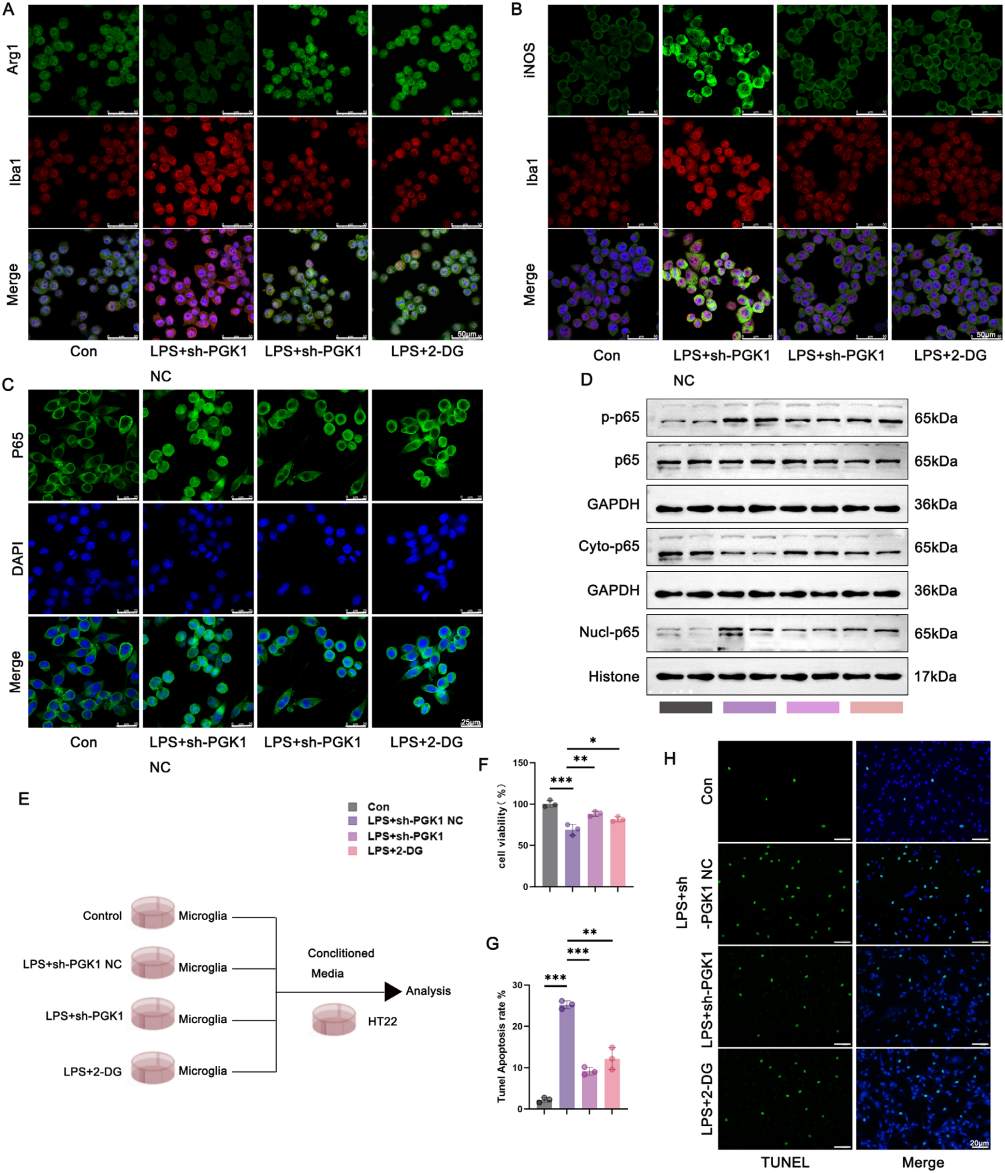
Targeting PGK1 suppresses inflammation and promotes neuronal survival by inducing M2 polarization in microglia. (A) Representative images of Iba1 and Agr1 immunofluorescence staining in each group of cells, scale bar = 50 μm. (B) Representative images of Iba1 and iNOS immunofluorescence staining in each group of cells, scale bar = 50 μm. (C) Representative images of P65 immunofluorescence staining in each group of cells, scale bar = 25 μm. (D) Protein expression levels of p‐p65, cytoplasmic p65 (Cyto‐p65), and nuclear p65 (Nucl‐p65) were determined by Western blot analysis. (E) Schematic diagram illustrating the conditioned medium experimental design, scale bar = 20 μm. (F) Cell viability was assessed across different groups using the CCK‐8 assay. (G) Quantitative analysis of TUNEL‐positive cells. (H) Representative fluorescent images of apoptotic cells detected by TUNEL staining. *n* = 3, *p < 0.05, **p < 0.01, ***p < 0.001, ns indicates no statistical significance.

Previous studies have demonstrated that PGK1 can suppress cellular inflammatory responses, but it remains unclear whether it affects microglial polarization. Immunofluorescence results showed that, compared to the Control group, LPS stimulation significantly increased Iba1 expression while decreasing Arg1 expression in microglia. In the PGK1 knockdown group, we observed significantly elevated Arg1 expression with strong colocalization with Iba1, indicating that reducing PGK1 promotes microglia polarization toward the neuroprotective M2 phenotype (Figure 4A). To investigate the effect of PGK1 knockdown on proinflammatory polarization, we examined iNOS expression. Immunofluorescence results revealed that under LPS‐induced M1 polarization conditions, Control group microglia exhibited strong iNOS signalling. However, in the PGK1 knockdown group, iNOS expression was significantly suppressed, suggesting that disrupting PGK1 function effectively inhibits neuroinflammatory responses (Figure 4B). Immunofluorescence analysis revealed that PGK1 knockdown significantly suppressed LPS‐induced nuclear translocation of p65 compared to the LPS + sh‐PGK1 NC group (Figure 4C). Consistently, Western blot analysis of subcellular fractions showed that upon LPS stimulation, PGK1 knockdown significantly decreased the levels of p‐p65 in whole‐cell lysates and p65 in the nucleus, while increasing p65 levels in the cytoplasm, relative to the LPS + sh‐PGK1 NC control (Figure 4D). These results indicate that PGK1 deficiency attenuates LPS‐induced p65 phosphorylation and inhibits its subsequent nuclear translocation, thereby effectively blocking the activation of the NF‐κB signalling pathway. To investigate the effects of microglia in different states on neuronal survival, we collected the culture medium from microglia under various treatment conditions and applied it to HT22 neurons (Figure 4E). Both CCK‐8 and TUNEL assays revealed that compared to medium from untreated microglia, medium from LPS‐treated microglia significantly reduced HT22 neuronal viability and increased the apoptosis rate of HT22 neurons. However, when using medium from PGK1‐knockdown microglia, LPS‐induced HT22 neuronal viability increased, and apoptosis rates were significantly reduced (Figure 4F–H). In contrast, the LPS + 2‐DG group consistently exhibited results similar to the control group in the aforementioned assays. These findings indicate that specific PGK1 knockdown significantly attenuates LPS‐induced inflammatory responses, demonstrating that PGK1 is a critical node in LPS‐triggered microglial inflammation.

### 3.5 PGK1 Regulates Microglial Polarization and Neuroinflammation by Driving Glycolysis

**FIGURE 5:**
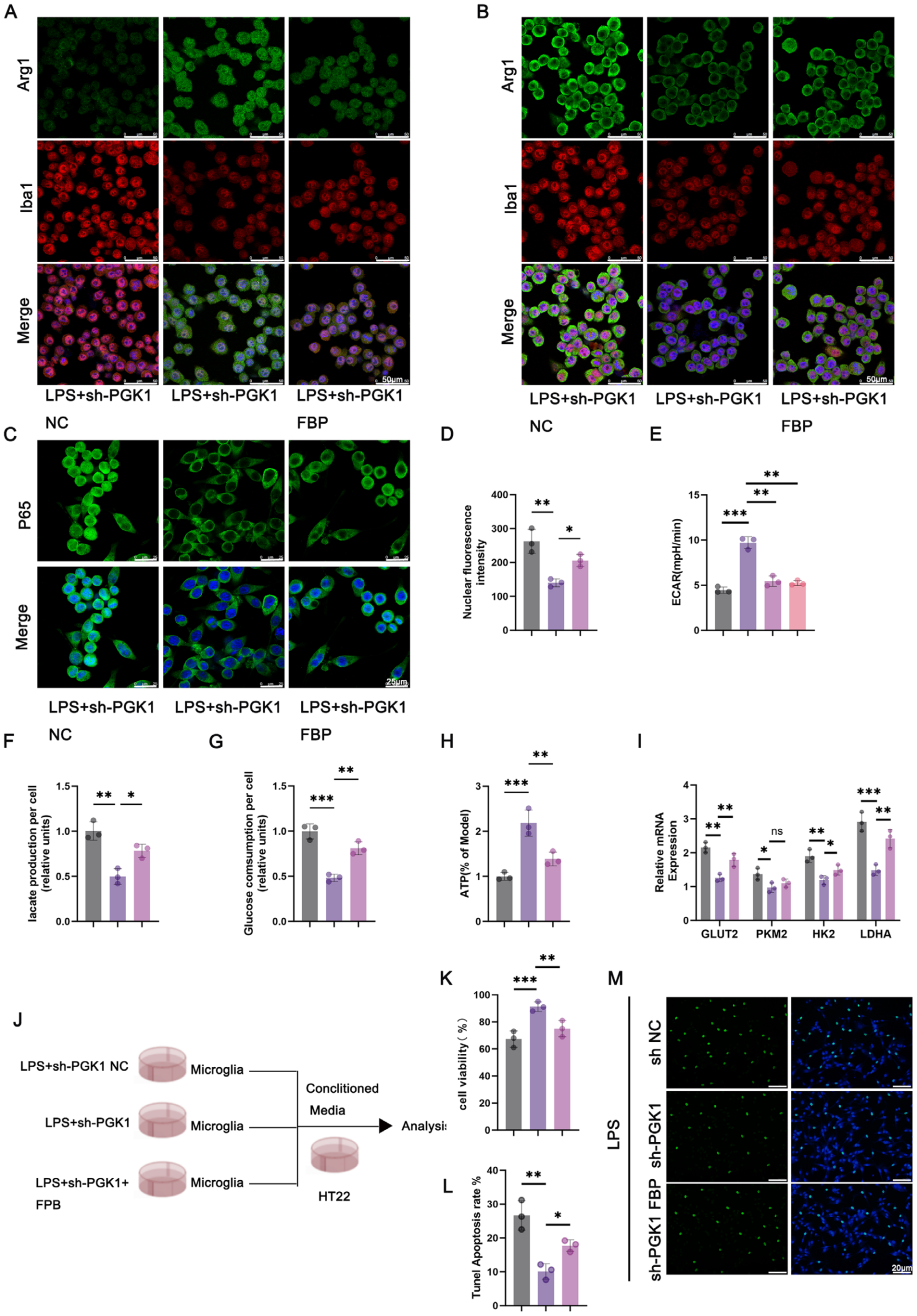
PGK1 regulates microglial polarization and neuroinflammation by driving glycolysis. (A) Representative images of Iba1 and Agr1 immunofluorescence in each group of cells, scale bar = 50 μm. (B) Representative images of Iba1 and iNOS immunofluorescence in each group of cells, scale bar = 50 μm. (C) Representative images of P65 immunofluorescence staining in each group of cells, scale bar = 25 μm. (D) Statistics of nuclear P65 fluorescence staining in each group of cells. (E) Baseline glycolysis levels in each group of cells. (F) Lactate content in cell culture medium across groups. (G) Glucose consumption per cell within each group. (H) Relative ATP content in cells across groups. (I) RT‐PCR detection of GLUT2, PKM2, HK2, and LDHA mRNA expression levels in cells across groups. (J) Schematic diagram of the conditioned medium experiment. (K) CCK‐8 assay for cell viability. (L) TUNEL‐positive cell counts statistics. (M) TUNEL assay for apoptosis detection, scale bar = 20 μm. *n* = 3, *p < 0.05, **p < 0.01, ***p < 0.001, ns indicates no statistical significance.

To further clarify whether PGK1 alleviates cellular inflammatory responses by driving glycolysis, we restored the glycolytic pathway using FBP. Immunofluorescence results revealed that, compared to the LPS + shPGK1 group, the LPS + shPGK1 FBP group exhibited enhanced Iba1 expression and significantly reduced Arg1 expression in microglia, indicating that restoring the glycolytic pathway inhibited the polarization of microglia toward the neuroprotective M2 phenotype (Figure 5A). Compared to the LPS + shPGK1 group, the LPS + shPGK1 FBP group exhibited significantly elevated iNOS expression in microglia and markedly increased P65 expression in cell nuclei, suggesting that restoring the glycolytic pathway also significantly intensified neuroinflammatory responses (Figure 5B–D). Additionally, we evaluated the glycolytic response in cells across all groups following FBP administration. Functional analysis revealed that compared to the LPS + shPGK1 group, the LPS + shPGK1 FBP group exhibited significantly elevated basal glycolytic activity in microglia (Figure 5E), suggesting that FBP partially restored the glycolytic pathway. This finding was further supported by biochemical assays, revealing that LPS + shPGK1 FBP‐treated microglia exhibited both markedly increased glucose consumption and lactate production compared to the LPS + shPGK1 group (Figure 5F,G). ATP detection results revealed that compared with the LPS + shPGK1 group, the LPS + shPGK1 FBP group exhibited significantly reduced ATP content in microglia (Figure 5H), suggesting that restoration of the glycolytic pathway substantially impacted ATP production. RT‐PCR results revealed significantly elevated mRNA levels of GluT2, PKM2, HK2, and LDHA in the LPS + shPGK1 FBP group compared to the LPS + shPGK1 group, suggesting that FBP may restore cellular metabolic reprogramming (Figure 5I). We further investigated the effect of FBP on neuronal survival in LPS‐stimulated PGK1‐knockdown microglia. Conditioned medium from microglia under different conditions was collected and applied to HT22 neurons. Dual CCK‐8 and TUNEL assays revealed that compared to conditioned medium from LPS + shPGK1‐treated microglia, conditioned medium from FBP‐treated microglia significantly reduced HT22 cell viability and increased overall apoptosis (Figure 5J–M). These findings indicate that PGK1 regulates microglial polarization and neuroinflammation by driving glycolytic pathways.

### 3.6 Specific Knockdown of PGK1 in Microglia Improves Isoflurane‐Induced Cognitive Impairment in Aged Mice

**FIGURE 6:**
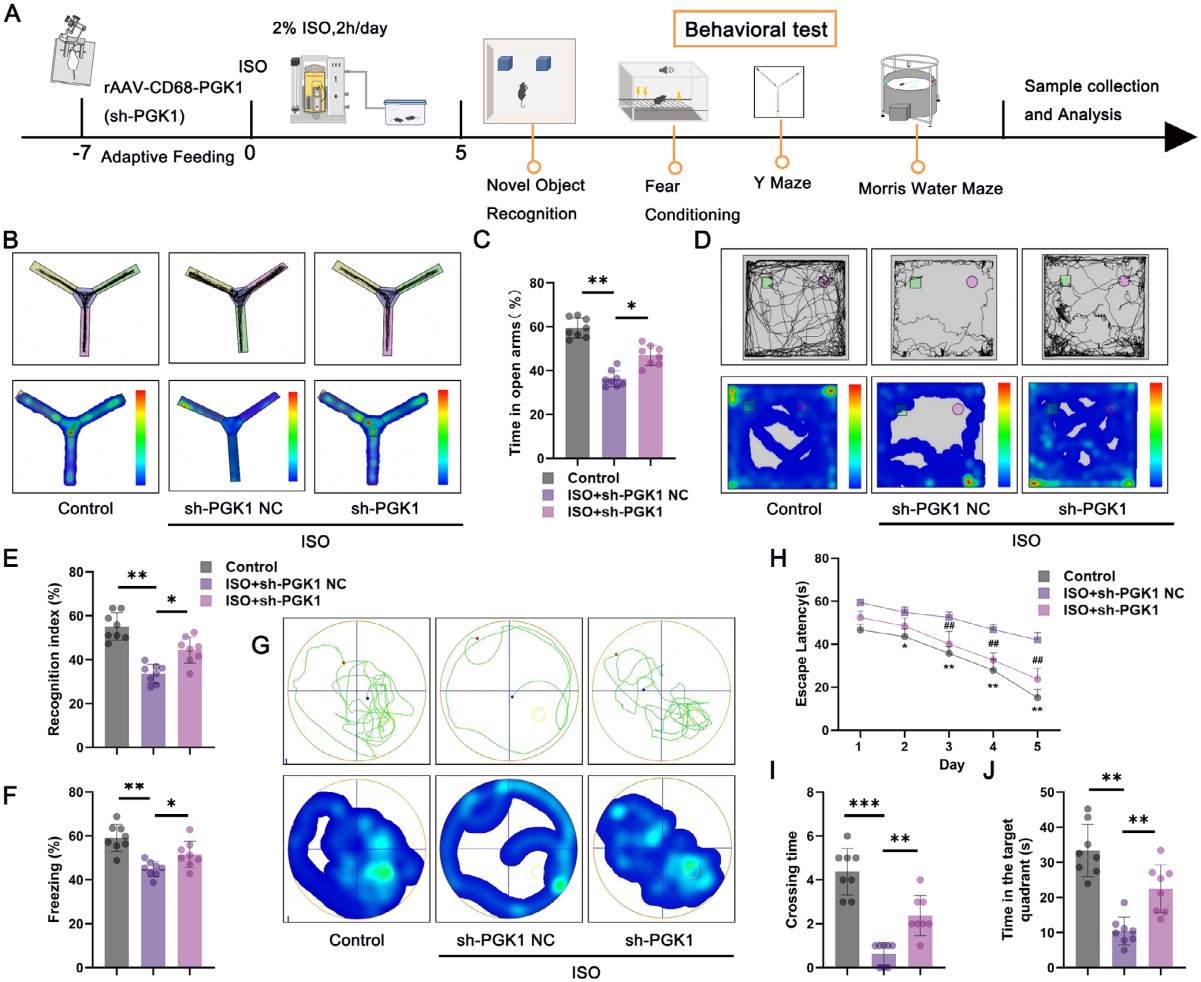
Specific Knockdown of PGK1 in Microglia Improves Isoflurane‐Induced Cognitive Impairment in Aged Mice. (A) Experimental design flowchart. (B, C) Mouse Y‐Maze experimental trajectories and statistics. (D, F) NOR mouse trajectories and statistical results. (G) MWM experimental mouse swimming trajectories. (H) MWM experimental mouse latency times. (I) MWM experimental mouse platform crossing counts. (J) MWM experimental mouse dwell time in target quadrants. *n* = 8, *p < 0.05, **p < 0.01, ***p < 0.001.

Prior to the experiment, we first treated the relevant mice with rAAV‐CD68‐PGK1 (sh‐PGK1). The mice underwent 1 week of acclimation, followed by ISO treatment and behavioural testing consistent with the method described in Figure 1A (Figure 6A). Y‐Maze results revealed that mice in the ISO + sh‐PGK1 group exhibited significantly prolonged entry times into the novel arm compared to the ISO + sh‐PGK1 NC group (Figure 6B,C), indicating that PGK1 knockdown restores ISO‐induced spatial memory deficits in mice. NOR results showed that compared with the ISO + sh‐PGK1 NC group, mice in the ISO + sh‐PGK1 group exhibited significantly longer exploration time toward novel objects and significantly increased exploration time toward familiar objects (Figure 6D). Further statistical analysis revealed that the discrimination index in the ISO + sh‐PGK1 NC group was significantly lower than that in the ISO + sh‐PGK1 group (Figure 6E), suggesting that PGK1 knockdown significantly protected object recognition memory in mice. CFCT results revealed that compared to the ISO + sh‐PGK1 NC group, mice in the ISO + sh‐PGK1 group exhibited a significantly prolonged freezing time ratio (Figure 6F), suggesting that PGK1 knockout attenuates ISO‐induced impairment of contextual associative memory in mice. The Morris water maze experiment investigated the effects of PGK1 knockout on isoflurane‐induced cognitive impairment in aged mice. Swimming trajectory results showed that mice in the ISO + sh‐PGK1 group exhibited significantly more platform quadrant trajectories compared to the ISO + sh‐PGK1 NC group (Figure 6G). Pre‐training results indicated that the latency to reach the platform from day 3 to day 5 was significantly reduced in the ISO + sh‐PGK1 group compared to the ISO + sh‐PGK1‐NC group, suggesting improved learning and memory capacity (Figure 6H). Second, compared with the ISO + sh‐PGK1 NC group, mice in the ISO + sh‐PGK1 group exhibited significantly increased crossings of the platform location and longer dwell time in the target quadrant, indicating enhanced memory retention (Figure 6I,J). These findings suggest that targeting PGK1 alleviates ISO‐induced cognitive impairment in mice.

### 3.7 Targeting PGK1 Protects Hippocampal Neurons From Isoflurane‐Induced Injury by Suppressing Neuroinflammation

**FIGURE 7:**
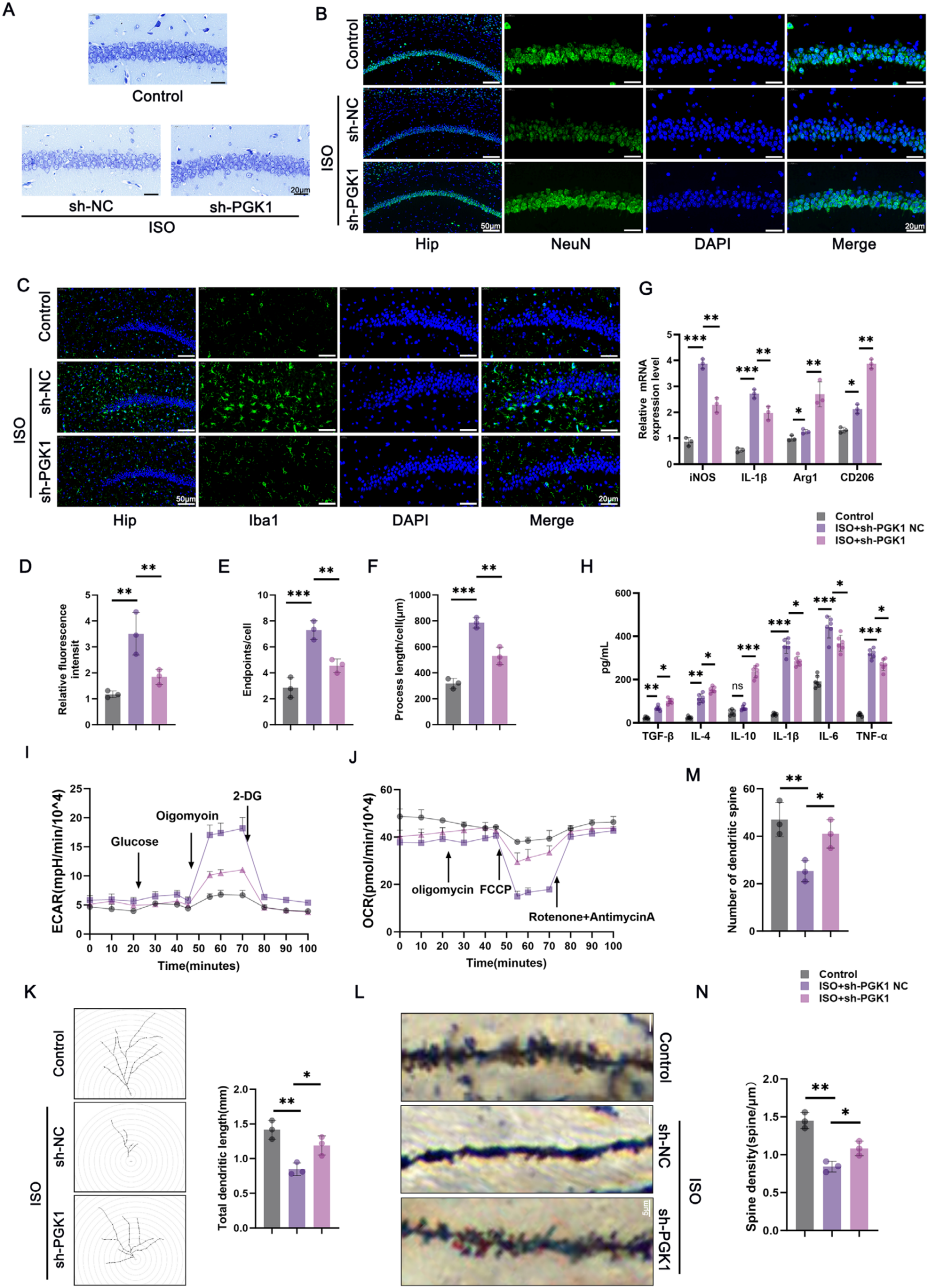
Targeting PGK1 protects hippocampal neurons from isoflurane‐induced injury by suppressing neuroinflammation. (A) Effect of PKG1 knockout on microglial morphology following ISO treatment, scale bar = 20 μm. (B) Representative images of Hip and NeuN immunofluorescence in cells from each group, scale bar = 20 μm. (C) Representative images of Hip and IBA1 immunofluorescence in cells from each group, scale bar = 20 μm. (D) Relative fluorescence intensity of ba1. (E) Number of cellular processes per microglia in each group. (F) Total length of all branches per microglia in each group. (G) RT‐PCR detection of mRNA expression levels for iNOS, IL‐1β, Arg1, and CD206 in hippocampal cells. (H) ELISA detection of cytokine expression levels for TNF‐β, IL‐4, IL‐10 in hippocampal cells. (I) Results of the glycolytic stress assay. (J) Results of the Seahorse XF Cell Mito Stress Test. (K) Sholl analysis of hippocampal neurons. (L) Representative images of dendritic spines in hippocampal neurons, scale bar = 5 μm. (M) Quantitative analysis of dendritic spine density. (N) Quantitative analysis of total dendritic spine number. *n* = 3, *p < 0.05, **p < 0.01, ***p < 0.001.

Previous studies have established that ISO induces neuronal damage in mice, but the role of PGK1 in this process remains unclear. Nissl staining revealed that, compared to the ISO + sh‐PGK1 NC group, ISO + sh‐PGK1 neurons exhibited compact, plump cell bodies, and abundant dark Nissl bodies within the cytoplasm (Figure 7A), suggesting that PGK1 knockdown mitigates ISO‐induced neuronal injury. Immunofluorescence analysis revealed stronger NeuN signalling and weaker Iba1 signalling in the hippocampus of the ISO + sh‐PGK1 group compared to the ISO + sh‐PGK1 NC group (Figure 7B–D). This indicates that PGK1 knockdown mitigates both ISO‐induced neuronal damage and immune responses in the hippocampal region. Statistical analysis of glial cell process and total branching length revealed that compared to the ISO + sh‐PGK1 NC group, the ISO + sh‐PGK1 group exhibited significantly increased glial cell processes, elongated processes, and markedly elevated total branching length. These morphological alterations indicate that glial cells remain inactivated (Figure 7E,F). RT‐PCR results revealed significantly reduced mRNA expression levels of iNOS, IL‐1β, Arg1, and CD206 in the ISO + sh‐PGK1 group compared to the ISO + sh‐PGK1 NC group, suggesting that PGK1 knockout mitigates ISO‐induced increases in intracellular inflammatory levels (Figure 7G). Furthermore, we measured the expression of key inflammatory cytokines (TNF‐β, IL‐4, IL‐10, IL‐1β, TNF‐α) by ELISA. The results showed that PGK1 knockdown significantly attenuated the ISO‐induced upregulation of these inflammatory factors compared to the ISO + sh‐PGK1 NC group (Figure 7H). The ECAR kinetics curve shows that glycolytic activity increases following ISO stimulation; knockdown of PGK1 significantly reduces ECAR values, suggesting that PGK1 is involved in regulating ISO‐induced glycolytic metabolic reprogramming (Figure 7I). The OCR kinetics curve results indicate that mitochondrial respiration increases following ISO stimulation; knockdown of PGK1 significantly restores mitochondrial respiration that had been reduced following ISO stimulation (Figure 7J). We next examined whether PGK1 knockdown could rescue the neuronal structural deficits. Sholl analysis revealed that neurons in the ISO + sh‐PGK1 group exhibited a significant increase in dendritic branching and total length relative to the ISO + sh‐PGK1 NC controls (Figure 7K). Consistent with this structural recovery, immunostaining demonstrated a marked increase in both the density and number of dendritic spines in the ISO + sh‐PGK1 group, indicating that PGK1 knockdown mitigated ISO‐induced synaptic damage (Figure 7L–N). Collectively, these findings suggest that knocking down PGK1 alleviates neuronal structural impairment and synaptic loss by suppressing microglial activation and neuroinflammation.

## 4 Discussion

We found that upregulation of PGK1 expression in ISO animal models and microglial inflammatory models promoted increased cellular glycolytic flux, thereby triggering neuroinflammation and ultimately leading to cognitive impairment in mice. This study confirms that PGK1 serves as a pivotal hub linking microglial glucose metabolism and inflammatory function, revealing a novel mechanism for ISO‐induced cognitive dysfunction in mice. These findings hold significant clinical implications for the precise diagnosis and treatment of cognitive impairment in mice.

The findings reveal that ISO impaired mice's short‐term working memory, spatial learning and long‐term memory, novel object recognition ability, and hippocampal‐dependent episodic memory. This confirms that ISO stimulation damages cognitive function in mice at the behavioural level. Previous studies have corroborated these results, and postoperative cognitive impairment remains a key concern for clinicians [30, 31]. Secondly, morphological studies also indicate that following ISO stimulation, neurons in the mouse hippocampus exhibit loosened and shrunken cell bodies, presenting a damaged‐like state. Cognitive disorders such as Alzheimer's disease, Parkinson's disease, and Huntington's disease have confirmed that progressive damage (oxidative stress, inflammation) and death of brain neurons constitute key mechanisms underlying the onset of cognitive diseases, consistent with our findings [32, 33, 34].

Subsequently, we observed that ISO stimulation caused damage to hippocampal neurons, enhanced immune responses, and simultaneously activated microglia. RT‐PCR analysis revealed elevated mRNA expression levels of iNOS, IL‐1β, Arg1, and CD206 following ISO stimulation. Furthermore, levels of inflammatory cytokines including TNF‐β, IL‐4, IL‐10, IL‐6, IL‐1β, and TNF‐α were significantly increased. Additionally, multiple studies have demonstrated persistently elevated levels of inflammatory cytokines in cognitive disorders associated with neurological injury [35, 36, 37, 38]. Under physiological conditions, neurons preferentially utilize oxidative phosphorylation to efficiently generate large amounts of ATP. In contrast, activated microglia employ glycolysis as their primary metabolic pathway during injury [39, 40]. Studies have revealed impaired glucose metabolism in the brains of patients with Alzheimer's disease and mild cognitive impairment [41, 42]. Glycolysis is no longer merely a simple energy metabolism pathway; it has become a pivotal hub for understanding the pathophysiology of neural injury and developing novel therapies.

PGK1, as a key protein kinase in glycolysis, participates in regulating neuronal excitability, synaptic plasticity (the basis of learning and memory), and circadian rhythms. Multiple findings confirm that ISO stimulation enhances PGK1 expression in microglia. We hypothesize that PGK1 may promote glycolysis, thereby inducing glial cells to polarize toward the pro‐inflammatory M1 phenotype and ultimately trigger neuroinflammation. To elucidate the role of PGK1 in ISO‐induced cognitive impairment in mice, we knocked out PGK1 in glial cells. Our findings revealed that PGK1 knockdown significantly reduced the LPS‐induced cellular glycolytic response, accompanied by decreased glucose consumption and lactate production, while cellular ATP levels increased. Additionally, mRNA levels of genes associated with metabolic reprogramming—GluT2, PKM2, HK2, and LDHA—were also downregulated, further confirming that reduced PGK1 expression suppresses LPS‐induced abnormal glycolysis in microglia. Cao et al. found that inhibiting PGK1 alleviates ischemia–reperfusion‐induced glycolysis, consistent with our findings [25]. Conversely, other studies indicate that enhancing PGK1 activity and glycolytic activity can alleviate the progression of neurodegenerative diseases [43]. Novel PGK1 activators can also serve as neuroprotective agents by inhibiting apoptosis [44]. We hypothesize that the contradictory outcomes of PGK1 regulation may correlate with glycolytic levels. Furthermore, in our study, reducing PGK1 significantly decreased LPS‐induced inflammatory mediators, providing additional evidence for PGK1's protective role in microglia.

Research has shown that reducing PGK1 expression can mitigate neuroinflammatory responses induced by ischemia–reperfusion injury, a finding consistent with our study [25]. Concurrently, PGK1 mediates inflammasome activation through NLRP3 phosphorylation, independent of its glycolytic activity [45]. Furthermore, PGK1 deficiency attenuates excessive inflammatory responses in acute lung injury, further clarifying PGK1's role in inflammation [26]. On the other hand, PGK1 also regulates the balance between pro‐inflammatory and anti‐inflammatory cytokines through interactions with other proteins [46]. In our study, we additionally found that LPS stimulation induces the M1 pro‐inflammatory phenotype in microglia, while inhibiting PGK1 promotes their conversion to the M2 anti‐inflammatory phenotype. Studies indicate that reducing PGK1 can inhibit M1 polarization of microglia by decreasing glycolysis [25]. Zhu et al. found that PGK1 promotes M1 macrophage polarization and induces pyroptosis by regulating NLRP3 in an acute lung injury model [26]. More importantly, we used FBP to demonstrate that PGK1 promotes M1 polarization of microglia by driving glycolytic regulation, thereby triggering neuroinflammation and potentially causing neuronal injury and cognitive impairment via the NF‐κB pathway. Conditional medium experiments further demonstrated that reducing PGK1 expression in microglia also mitigated LPS‐induced neuronal damage, a process mediated by PGK1 through regulation of glycolysis. Finally, in vivo specific knockout of PGK1 significantly alleviated ISO‐induced cognitive impairment, microglial polarization, inflammatory response, and neuronal damage in mice.

In addition, the limitations of this study are as follows: (1) Only aged male mice were used; gender differences in female mice were not assessed. Although estrogen levels are already very low in aged females, sex chromosomes and other sex hormones may still lead to different phenotypes. (2) Cognitive function was observed only in the short term (≤ 7 days) following anaesthesia, without long‐term follow‐up; consequently, it remains unclear whether ISO‐induced cognitive impairment is short‐term and reversible or long‐term and persistent. Future studies should include female mice and extend the observation period to verify the generalizability and persistence of the findings of this study.

**FIGURE 8:**
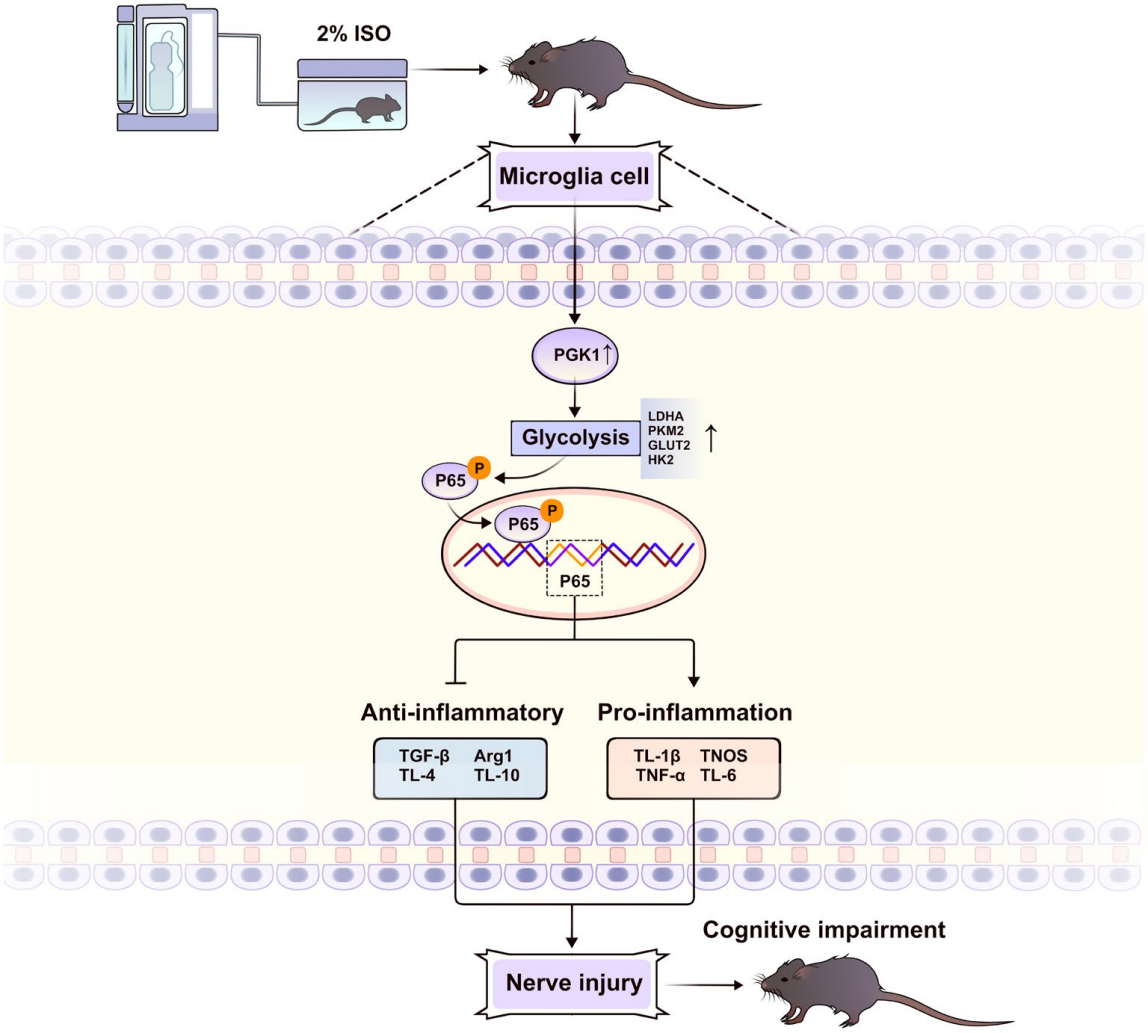
Isoflurane links PGK1 upregulation to cognitive deficits in mice via microglial metabolic reprogramming and neuroinflammation.

In summary, isoflurane (ISO) enhances glycolytic flux by activating NF‐κB to upregulate PGK1 and glycolytic enzymes, thereby reprogramming glycolysis in microglia and leading to cognitive impairment in aged mice (Figure 8).

Targeting PGK1 may offer a novel metabolic intervention strategy for alleviating cognitive impairment and neuroinflammation in these animals. Targeting PGK1 may offer a novel metabolic intervention strategy for alleviating cognitive dysfunction and neuroinflammation in these animals.

## Author Contributions

**Jiahao Guo:** conceptualization, methodology, software, writing – original draft. **Yujie Ma:** investigation, visualization. **Ruiyun Guo:** investigation, visualization. **Kai Yuan:** investigation, visualization. **Huaying Hu:** conceptualization, methodology, writing – original draft, data curation. **Shuntao Jiao:** software, visualization. **Yajun Liu:** formal analysis, data curation. **Dongliang Zhang:** writing – review and editing, resources, supervision, funding acquisition. **Dengfa Zhao:** validation, formal analysis. **Zhuo Ren:** conceptualization, methodology, investigation, writing – original draft. **Chenyang Qiu:** validation, visualization. **Jun Ma:** writing – review and editing, project administration, supervision. **Mianwang He:** writing – review and editing, project administration, supervision. **Yang Zeng:** writing – review and editing, supervision, funding acquisition, resources.

## Funding

This study was supported by the National Key R&D Program of China (2022YFA1106300); the National Natural Science Foundation of China (82370108); and the Noncommunicable Chronic Diseases‐National Science and Technology Major Project (2023ZD0507900).

## Ethics Statement

All experimental procedures were approved by the Animal Welfare and Ethics Committee of Beijing MDKN Biotechnology Co. Ltd. and were strictly conducted in accordance with the Beijing Animal Control Committee's Guidelines for the Care and Use of Laboratory Animals (MDKN‐2024‐210).

## Conflicts of Interest

The authors declare no conflicts of interest.

## Supporting information


**Figure S1:** PGK1 exacerbates neuroinflammation by driving glycolytic reprogramming and impairing energy metabolism (A) Number of cell processes in individual microglia in panel C (F) Total length of all branches in individual microglia in panel C (C) Glucose consumption per cell within each group (D) Lacate production per cell within each group (E) Glycolysis capacity per cell within each group (F) Glycolysisper cell within each group (G,I) Immunofluorescence staining for NEUN and PGK1 in each group of cells, scale bar = 20 μm (H,J) Immunofluorescence staining for GFAP and PGK1 in each group of cells, scale bar = 20 μm (L,K) Immunofluorescence staining for Iba1 and PGK1 in each group of cells, scale bar = 20 μm.*n* = 3, **p < 0.01, ***p < 0.001, ns indicates no statistical significance.

## Data Availability

The data that support the findings of this study are available on request from the corresponding author. The data are not publicly available due to privacy or ethical restrictions.
