# Supplementary material for: PGK1 Drives Glial Glycolytic Reprogramming to Mediate Isoflurane‐Induced Cognitive Impairment in Aged Mice
Source: J Cell Mol Med. 2026 Jul 2;30(13):e71276. doi: 10.1111/jcmm.71276 (PMC13329126; doi:10.1111/jcmm.71276)
Supplement: Supplementary file 1 — Figure S1: PGK1 exacerbates neuroinflammation by driving glycolytic reprogramming and impairing energy metabolism (A) Number of cell processes in individual microglia in panel C (F) Total length of all branches in individual microglia in panel C (C) Glucose consumption per cell within each group (D) Lacate production per cell within each group (E) Glycolysis capacity per cell within each group (F) Glycolysisper cell within each group (G,I) Immunofluorescence staining for NEUN and PGK1 in each group of cells, scale bar = 20 μm (H,J) Immunofluorescence staining for GFAP and PGK1 in each group of cells, scale bar = 20 μm (L,K) Immunofluorescence staining for Iba1 and PGK1 in each group of cells, scale bar = 20 μm.n = 3, **p < 0.01, ***p < 0.001, ns indicates no statistical significance. [file JCMM-30-e71276-s001.docx]

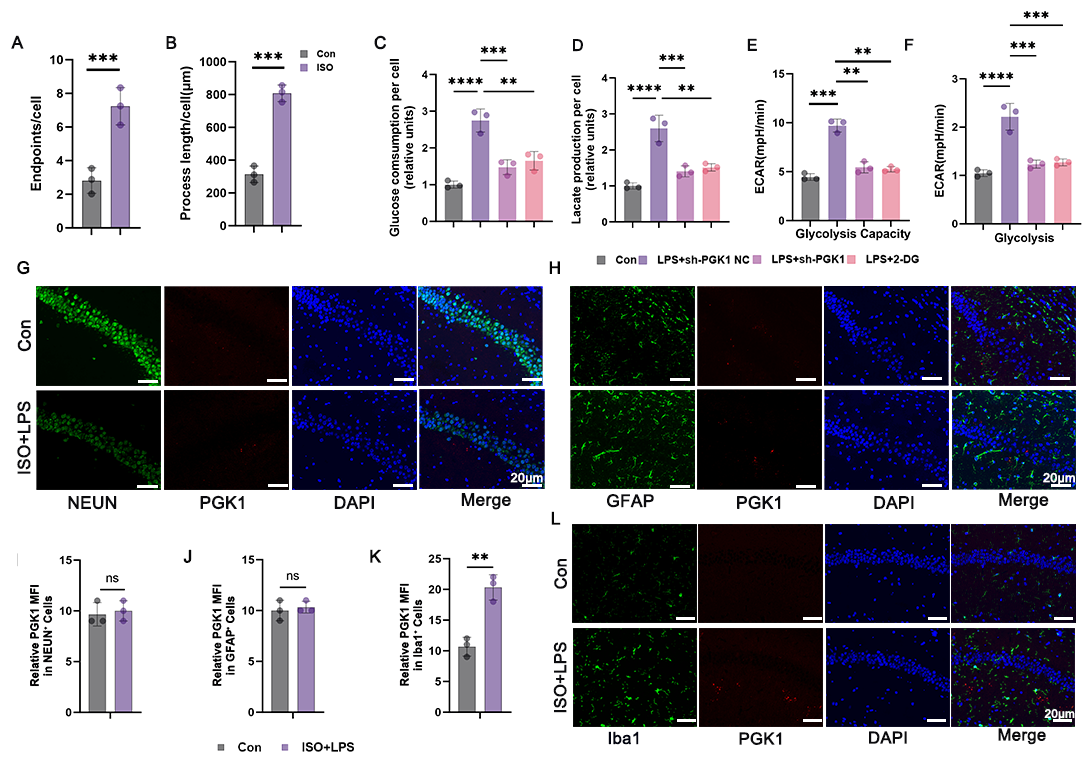


Figure. S1 PGK1 exacerbates neuroinflammation by driving glycolytic reprogramming and impairing energy metabolism. (A) Number of cell processes in individual microglia in panel C. (F) Total length of all branches in individual microglia in panel C. (C) Glucose consumption per cell within each group. (D) Lacate production per cell within each group. (E) Glycolysis capacity per cell within each group. (F) Glycolysisper cell within each group. (G,I) Immunofluorescence staining for NEUN and PGK1 in each group of cells, , scale bar = 20µm.(H,J) Immunofluorescence staining for GFAP and PGK1 in each group of cells, scale bar = 20µm.(L,K) Immunofluorescence staining for Iba1 and PGK1 in each group of cells, scale bar = 20µm.n=3, ***P*<0.01, ****P*<0.001, ns indicates no statistical significance.
